# Application of deep eutectic solvents in protein extraction and purification

**DOI:** 10.3389/fchem.2022.912411

**Published:** 2022-09-06

**Authors:** Hou Bowen, Rabia Durrani, André Delavault, Erwann Durand, Jiang Chenyu, Long Yiyang, Song Lili, Song Jian, Huan Weiwei, Gao Fei

**Affiliations:** ^1^ Zhejiang Provincial Key Laboratory of Resources Protection and Innovation of Traditional Chinese Medicine, Zhejiang A & F University, Hangzhou, China; ^2^ State Key Laboratory of Subtropical Silviculture, Zhejiang A & F University, Lin’an, Zhejiang, China; ^3^ Technical Biology, Institute of Process Engineering in Life Sciences II, Karlsruhe Institute of Technology, Karlsruhe, Germany; ^4^ CIRAD, UMR QUALISUD, Montpellier, France; ^5^ Qualisud, Univ Montpellier, Avignon Université, CIRAD, Institut Agro, Université de la Réunion, Montpellier, France; ^6^ Zhejiang Provincial Key Laboratory of Chemical Utilization of Forestry Biomass, College of Chemistry and Materials Engineering, Zhejiang A & F University, Hangzhou, Zhejiang, China

**Keywords:** deep eutectic solvent, protein, extraction, green solvent, biodegradability

## Abstract

Deep eutectic solvents (DESs) are a mixture of hydrogen bond donor (HBD) and hydrogen bond acceptor (HBA) molecules that can consist, respectively, of natural plant metabolites such as sugars, carboxylic acids, amino acids, and ionic molecules, which are for the vast majority ammonium salts. Media such as DESs are modular tools of sustainability that can be pointed toward the extraction of bioactive molecules due to their excellent physicochemical properties, their relatively low price, and accessibility. The present review focuses on the application of DESs for protein extraction and purification. The in-depth effects and principles that apply to DES-mediated extraction using various renewable biomasses will be discussed as well. One of the most important observations being made is that DESs have a clear ability to maintain the biological and/or functional activity of the extracted proteins, as well as increase their stability compared to traditional solvents. They demonstrate true potential for a reproducible but more importantly, scalable protein extraction and purification compared to traditional methods while enabling waste valorization in some particular cases.

## Introduction

In order to maintain the environmental balance, several sectors of industry, most notably the food industry, have started to use greener solvents as alternatives in their manufacturing processes rather than synthetic, petroleum-derived organic solvents (Ling and Hadinoto, 2022). Synthetic organic solvents are extensively used in foods, pharmaceuticals, and cosmetics for several purposes such as extraction and separation processes (Jesus and Filho, 2020). Depending on the polarity of the solvent selected, they have greater compatibility for hydrophilic or hydrophobic compounds. A number of these volatile solvents are obtained from fossil fuels and display several drawbacks to the environment like being toxic, non-degradable, and flammable (Fuad et al., 2021). Therefore, industrials are eager to shift to environmentally friendlier and greener solvent alternatives for the manufacturing of their products. Currently, some of these alternatives include supercritical fluids, ionic liquids (ILs), and deep eutectic solvents (DES). These solvents are less hazardous, environmentally less impactful, and consume less energy for their production and waste management (Anastas and Eghbali, 2009). Amongst these greener solvents, ILs and DESs are promising and well-known media of this new era of solvent engineering. ILs are non-flammable, have low vapor pressure, and are highly stable. In addition, they remain in the liquid state at a temperature below 100°C. However, due to their higher production cost, tedious recycling, and purification, their use is limited to the laboratory scale (Fuad et al., 2021). To cover such limitations, DESs are newly developed greener solvents that have much more significance with the aim of sustainability (Kudłak et al., 2015). They are, according to Kist et al., “cousins” of the ILs (Kist et al., 2020). DESs are eutectic mixtures of Lewis or Brønsted acids and bases, that is, hydrogen bond donors (HBDs) and hydrogen bond acceptors (HBAs). ILs and DESs have similar physical properties such as lower volatility, higher viscosity, and non-flammability and both have relatively good thermal stability. However, DESs are not completely like ILs as they can be partly or fully prepared from non-ionic compounds. This is in part the reason for their cost effectiveness, environmental friendliness, and degradability as they are made from naturally occurring metabolites. Given the superior possible number of combinations forming DESs, they are a versatile tool of sustainability that can be applied in various areas. Extraction and purification of proteins being topical and a currently investigated challenge as well for both academia and industry seemed logical that these two subjects merged into one. Being the greener solvent with greater interest, DESs represent a unique solution for a refinery in regard to a crucial need for sustainability. DESs are easy to prepare, and their supramolecular structures are often a good match for protein extraction, according to the three following conditions: affinity, solubility, and stability (Landa et al., 2020).

DESs are classified into four categories according to the general formula described in Table 1.

**TABLE 1:** General formula for the classification of DESs.

Type	General formula	Terms
Type I	Cat^+^X^−^ zMCl_x_	M = Zn, Sn, Fe, Al, Ga, and In
Type II	Cat^+^X^−^ zMCl_x_·yH2O	M = Cr, Co, Cu, Ni, and Fe
Type III	Cat^+^X^−^ zRZ	Z = CONH_2_, COOH, and OH
Type IV	MCl_x_ + RZ = MCl_x−1_^+^·RZ + MCl_x+1_^−^	M = Al and Zn and Z = CONH_2_ and OH

The most commonly used DES involved in protein studies belongs to type III and consists mostly of choline chloride (ChCl) as HBA and amines, amide, carboxylic acids, sugars, and polyols as HBD (Zhang et al., 2012a). The molar ratio composing them is an essential factor, as depicted in Table 2 so that after mixing the HBD and HBA in the appropriate molar ratio, the ions are paired with available hydrogen ions to form a mixture that is stable at a temperature below their respective melting points (Ling and Hadinoto, 2022). DESs were first introduced by Abbott et al. (2003) in which ChCl (mp = 302°C) was mixed with urea (mp = 133°C) to form a resulting mixture with a melting point of 12°C. This drastic decrease in the melting point was explained by the delocalization of electrons that occurs from the hydrogen bonding between halide ions and HBDs (Abbott et al., 2003).

**TABLE 2:** Molar ratios used for the preparation of common ChCl-based DESs.

Deep eutectic solvents	ChCl: HBD
HBA: HBDs	(Molar ratio)
ChCl: urea	1:2
ChCl: acetamide	1:2
ChCl: ethylene glycol	1:2
ChCl: glycerol	1:2
ChCl: xylitol	1:1
ChCl: sorbitol	1:1
ChCl: glucose: H_2_O	5:2:5
ChCl: sucrose: H_2_O	5:2:5
ChCl: xylose: H_2_O	5:2:5
ChCl: malic acid	1:1

**FIGURE 1:**
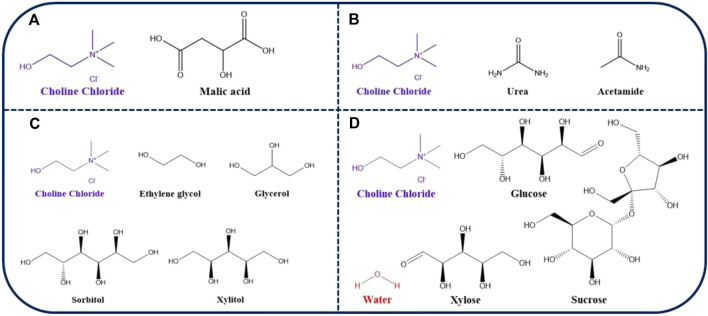
Chemical structures of the most used HBA (ChCl) and HBDs in order to form DESs commonly applied to protein extraction and purification with **(A)** ChCl: malic acid, **(B)** ChCl: urea, ChCl: acetamide, **(C)** ChCl: ethylene glycol, ChCl: glycerol, ChCl: sorbitol, ChCl: xylitol, **(D)** ChCl: glucose: H_2_O, ChCl: sucrose: H_2_O, and ChCl: xylose: H_2_O.

Type III is one of the simplest types of DES in its preparation, costing significantly less than other DESs and are highly biocompatible as they commonly use ChCl as HBA (Figure 1). ChCl is classified in the family of B vitamins and is a primary, ubiquitously occurring plant metabolite that is easily biodegradable, relatively cheap, and displays low toxicity. It is present in animal feed and food supplements (Kist et al., 2020). Due to their promising environmentally friendly nature, DESs are extensively used in the extraction and purification of proteins. Indeed, a number of researchers have worked on the extraction of proteins from biomass looking for superior extraction yields (Ling and Hadinoto, 2022).

Natural deep eutectic solvents (NADESs) are a concept first introduced by Choi et al. (2011), in which primary plant metabolites (sugars, acids, and amino acids) were used in order to form DESs that could be potentially identified in nature. They are also alternate solvents used for the extraction of proteins. They are far more environmental friendly due to their natural origin. It is thought that these natural solvents may be present in all organisms and involved in biosynthesis, solubility, and storage of hydrophobic metabolites in living organelles (Kist et al., 2020). These solvents are the most promising for the extraction and purification of proteins and can open new gateways for industrialization.

## Deep eutectic solvents as media for protein extraction

**FIGURE 2:**
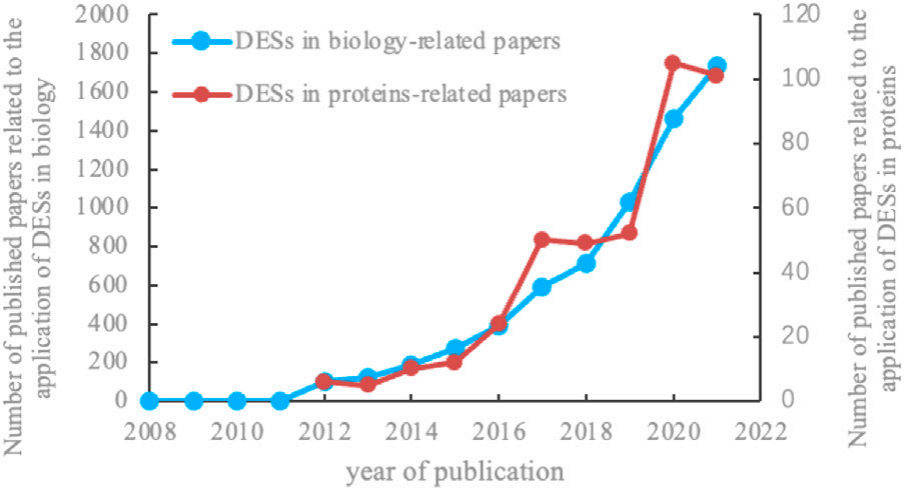
Publications involving DESs between 2008 and 2022 related to the general study of proteins in biology (blue) or specifically in extraction and purification (red) (published data from Web of Science).

Proteins are the fundamental unit of life that can be extracted from various sources like plants, animals, and microorganisms (Kumar et al., 2021). Investigations of efficient and sustainable protein extraction methods from various renewable biological sources are a growing area of interest in academia, as depicted in Figure 2 (Kaijia et al., 2015). Parallelly, the incorporation of high-quality protein in diet and their formulation in nutraceuticals has led the industry to search for effective but greener methods for protein extraction as well (Justino et al., 2014). In the following subparts, DES-assisted protein extraction that includes solid–liquid extraction and liquid–liquid extraction will be detailed. Solid–liquid extraction involves mixing solid components into a liquid medium, and liquid–liquid extraction involves separation or partitioning of two compounds into an immiscible one (Ling and Hadinoto, 2022).

### Deep eutectic solvent-mediated solid–liquid extraction

A remarkable example of this concept has been made in the work of Yue et al. (2021), in which ChCl was mixed with diols of various chain lengths for the extraction of oat protein (Table 3). Out of the nine different mixtures, they found that oat protein could be extracted with an extraction efficiency of up to 55.72% when using ChCl-1,4-butanediol/water. When reaching high solubility in the latter, better protein stability and foaming capacity were also enabled. Butanediol was shown to be the HBD responsible for facilitated extraction of oat protein. It was also observed that few DES mixtures could precipitate protein quickly, while binary mixtures took longer precipitation times. This mechanism may be due to the divergence and polarity of DESs which was advantageous for protein precipitation (Yue et al., 2021).

**TABLE 3:** Effect of DESs made with iso-alcohol of different chain hydrocarbon lengths on oat protein extraction yield.

S. no	Types of DES	Protein content	Protein extraction yield
1	ChCl: 1,2-ethanediol (1: 3)	54.69 % ± 3.55%	25.41% ± 0.63%
2	ChCl: 1,3-propanediol (1: 3)	48.88% ± 1.58%	23.77% ± 0.90%
3	ChCl: 1,4-butanediol (1: 3)	45.25% ± 2.55%	25.17% ± 0.79%
4	ChCl: 1,2-ethanediol: H_2_O (1: 2: 1)	51.29% ± 2.58%	20.55% ± 1.32%
5	ChCl: 1,2-ethanediol: H_2_O (1: 3: 1)	56.38% ± 0.69%	25.01% ± 0.55%
6	ChCl: 1,3-propanediol: H_2_O (1: 2: 1)	54.58% ± 1.18%	31.46% ± 1.07%
7	ChCl: 1,3-propanediol: H_2_O (1: 3: 1)	50.71% ± 3.16%	32.29% ± 0.91%
8	ChCl: 1,4-butanediol: H_2_O (1: 2: 1)	62.50% ± 1.38%	35.76% ± 1.31%
9	ChCl: 1,4-butanediol: H_2_O (1: 3: 1)	57.17% ± 3.01%	37.15% ± 0.85%

Convergently, Chen et al. (2021) mixed ChCl with glycerol for the extraction of soy protein and observed that DES-based extraction showed a yield higher by 10% than that of the acid-based precipitation method. Soy protein extracted by means of DESs also showed improved heat resistance and better hydrophobicity than native soy protein, indicating that DESs can enhance the physical properties of the proteins they solubilize, and thus, they can be further used as functional ingredients. Lin et al. (2021) extracted protein from bamboo shoots using an acidic DES based on ChCl and levulinic acid. They compared the extraction efficiency obtained with this mixture to a method using an aqueous solution of sodium hydroxide. The experiment resulted in an extraction yield enhanced by 60%, which ultimately provides another proof of DESs’ suitability for the extraction of proteins from renewable raw biomasses (Chen et al., 2021).

Wahlstrom et al. (2017) used potassium and sodium salts of both formate and acetate for the production of DESs in combination with urea as HBD, in order to extract protein from brewer´s spent grain originating from barley grain husk. They measured that NaOAc: urea (1:2) yielded a 79% protein extraction recovery while being composed of naturally occurring metabolites and could be assimilated to the NADES definition. In a need to develop formulations suitable for human consumption, NADESs are indeed a promising alternative that remain effective during the extraction process and could be applied widely in the food industry (Wahlström et al., 2017). Still with an aim of waste valorization, Rodrigues et al. (2021) mixed betaine and propylene glycol in a 1:3 ratio for the extraction of proteins from sardine processing residues. When sardines enter the processing line in a factory, its head is mechanically removed, and entrails and other parts will be separated from the commercialized end-product, thus generating a significant amount of waste along the way. They found out that DES-based extraction yielded up to a concentration of 162.2 mg/g protein and compared their results to a simple water-based extraction method. Surprisingly, they found a great number of hydrophobic amino acids such as alanine, isoleucine, leucine, and valine to be suitable to form relatively less polar DESs that were in the end, a good match to extract proteins from sardine processing wastes. The resulting DES extracts also showed superior antioxidant and antimicrobial activities in comparison to water extracts. It was suspected that the presence of hydrophobic DES components facilitates the interaction between the antimicrobial peptides with the cell membrane of the tested microorganisms. This feature demonstrates the usefulness of low polarity DESs in comparison to a highly polar solvent such as water. This method, however, requires in-depth investigations to understand better the biological effects of protein-enriched DES extracts and ensure their safety for human consumption (Rodrigues et al., 2021).

Oppositely, Hernández-Corroto et al. (2020) used a highly polar and hydrophilic DES made of ChCl and acetic acid for the extraction of protein from pomegranate peels assisted by ultrasonication. This method resulted in a protein content of 19.2 mg/g and was compared to a simple liquid extraction method. The resulting high protein content showed high antihypertensive effects.

Finally, Liu et al. (2017) mixed various HBAs such as choline chloride (ChCl), glycine, betaine, alanine chloride, acetylcholine chloride, and nicotinic acid with PEG200 at a ratio of 1:4 for protein extraction from pumpkin seeds. They found that aqueous PEG200: ChCl and aqueous PEG200: glycine showed excellent extraction efficacies. They also explored isoelectric point-ethanol-DES ternary co-precipitation as a method for protein recovery. They mixed PEG200-based DES with four-fold the volume of ethanol at pH 4.5 with the addition of 1 M HCl and separated the protein precipitate by centrifugation. The resulting precipitate was washed with water at pH 4.5 and centrifuged again. This straightforward approach combined isoelectric point-ethanol settlement, and DES self-settlement precipitation allowed to obtain a large amount of protein precipitate, thus reaching a remarkable settlement rate of 93.8% (Table 4).

**TABLE 4:** Methods used for protein precipitation in DESs using various protein sources.

Source of proteins	Protein precipitation method	Protein sedimentation yield	Reference
Pumpkin seed	Ternary co-precipitation	97% ± 0.8%	Liu et al. (2017)
	Four times the volume of ethanol precipitation	92% ± 1.08%	
	Isoelectric point precipitation	77% ± 2.8%	
	PEG 200-based DES self-precipitation	61% ± 1.3%	
Evening primrose cake	Water precipitation	19%	Grudniewska et al. (2018)
Rapeseed cake		34%	

Throughout these various studies, it was shown that DES-based extraction could be coupled with other techniques such as ultrasonication to improve the extraction efficiency of proteins and opens a promising horizon for development at the industrial scale, more notably for waste valorization in biorefineries. Nowadays, PEG-based DES extraction is getting more commonly used as it stabilizes the extracted proteins and is, in addition, approved by the FDA (Food and Drug Administration) (Morgenstern et al., 2017). This opens up many possibilities for more sustainable developments for sectors such as the pharmaceutical and agronomical industries. It can be argued that more research needs to be carried out to prove the scalability and practicability of PEG-based DES at the ton scale, albeit they demonstrate high affinity to proteins and are used as a precipitant to facilitate their crystallization (Kumar et al., 2009). A good hint is that in almost all methods depicting solid–liquid extraction covered in this study, water is used to reduce the viscosity of DESs and thus helps the extraction process in both efficiency and practicability. A higher viscosity in DES is actually related to a high number of hydrogen bonds, and it can hinder protein solubilization processes in some extreme cases. However, a proper amount of water should be used so that it may not disturb hydrogen bond interactions between the components of a DES as, oppositely, an excessive amount of water will end up wasting the useful properties of the DES (Ling et al., 2020).

### Deep eutectic solvent-mediated liquid–liquid extraction

Apart from solid–liquid extraction of proteins, liquid–liquid extraction of proteins has also been extensively studied and often comes along aqueous two-phase systems (ATPS) as it ensures, in some cases, an efficient recovery of the targeted protein (Pei Xu, Zheng, Du, Zong, & Lou, 2015). This system is formed by mixing a water-soluble polymer with another polymer or inorganic salt above the critical concentration (Hatti-Kaul, 2000), for example, PEG-salt-water systems and ethylene oxide–propylene oxide or copolymer–polyoxyethylene detergent systems. Xu et al. (2015b) mixed ChCl and glycerol to form an ATPS with a salt solution for the extraction of BSA (bovine serum albumin). It was shown that 98.16% of BSA was actually extracted from the DES-phase, and further investigation also demonstrated that there was no change in the conformation of protein by UV-vis, FT-IR, and circular dichroism. They also showed that this protein separation process is not dependent on electrostatic interactions; indeed, it rather triggers the formation of protein aggregates as evidenced by transmission electron microscopy. Another study reported the formation of aggregates between DES and BSA for the uptake of protein by DES-based ATPS. This protein uptake by DES is facilitated by hydrogen bonding, salting out, and hydrophobic interactions, which are all inherent to DES’s nature (Xu et al., 2015b).

Xu et al. (2019) extracted lysozyme using DES-based ATPS and found that 98% of the latter was contained in the DES-phase while maintaining 91.7% of its activity. Similarly, Meng et al. (2019) used a mixture of tetrabutylammonium chloride, polypropylene glycol 400 as an ATPS, and L-proline: xylitol as DES for the extraction of chymotrypsin based on the aforementioned principle. Superior extraction efficiencies of up to 97.3% could be reached, demonstrating the tremendous potential of DES-based ATPS.

### Limitations to deep eutectic solvent-assisted extraction of proteins

There are numerous chemicals that can fulfill the roles of HBD and HBA inside a DES, and thus, many combinations allowing a great number of DESs are possible. However, not every DES is suitable for protein extraction, and a universal method for their selection based on the desired application is lacking. Therefore, one needs to screen for suitable DESs that facilitate protein extraction of a given biomass, thus demanding extensive time and resources (Smith et al., 2014). Inherently to DESs, temperature affects drastically the viscosity and conductivity of the latter. At room temperature, the viscosity of a DES is generally higher than that of water, but it will decrease with increasing temperature, and on the contrary, its conductivity will increase with the increasing temperature (Lores et al., 2016). Overall, these aspects can represent serious challenges of practicability and scalability that can repel applications in the industry.

Despite the reportedly high efficiencies of extraction that were previously compiled, the limitation to this system concerns the recovery and isolation of proteins extracted in the DES phase. This step is known as the back extraction and is crucial for both DES recycling and protein separation. Protein recovery from DES is very slow due to high interfacial mass transfer resistance (Kaijia et al., 2015). Xu et al. performed back extraction by changing the concentration of salt, practically speaking it consist of mixing DESs with a freshly prepared salt solution. In doing so, they recovered only 32.9% of the protein contained in DES and used ethanol combined with a saline solution for the recovery. Keeping in view this limitation, the protein back extraction methods and the recovery of DESs need to be further improved in order to make them attractive for industries (Li et al., 2016). Nonetheless, their uniqueness and innovative aspects among extraction media have drawn the attention of the scientific community, and they have been applied to the processing of many original renewable sources of proteins.

## Deep eutectic solvents as useful modular tools for extraction of valuable animal and plant proteins: Specific case studies

A wide range of raw biomasses have been identified in the previous literature as containing proteins and were compatible for a DES-mediated extraction (Table 4). Many of these proteins possess interesting properties and not only as an added value in nutrition but more importantly in their inherent functionality. Interesting cases have been made for rapeseed and *Oenothera biennis* oils that both reduce blood lipid and possess the anti-arteriosclerosis activity. However, the production of these oils generates a large amount of deoiled cake waste. Thus, extracting compounds from this process waste is economically viable for the industry due to its valorization. Grudniewska et al. (2018) extracted protein from rapeseed and *O. biennis* cakes with ChCl: glycerol (1:2) and obtained a protein-rich precipitate by adding water as an anti-solvent to the protein extraction solution.

More than simply extracting plant proteins, DESs are also involved in the extraction of protein from animal hair, that is, rabbit hair and wool fiber. As considerable amounts of wool waste are produced every year globally by the textile industry, a tremendous environmental challenge arises. Herein, the concerned protein is keratin, a reactive, biocompatible, and biodegradable material that can be recovered when recycling wool. Wang et al. (2018) dissolved the latter in a DES composed of ChCl and oxalic acid. They extracted and separated keratin with molecular weights of 3.8–5.8 kDa and presented a high amount of serine, glutamic acid, cysteine, leucine, and arginine. Furthermore, using this same acidic DES, they dissolved rabbit hair with protein solubility greater than 70%, which was before unheard of. However, extraction from other protein-rich animal tissues possessing, that is, high nutritional or functional values using DESs has not been explored further but offers promising horizons (Wang and Tang, 2018).

## Deep eutectic solvent-mediated protein purification

Preparation of pure protein is of prime importance for research, the pharmaceutical industry, and other sectors of industry in order to commercialize safe products for use or actual consumption. Conventional ways of protein purification include alkali, ammonium sulfate or acetone precipitation; salting-out, ion exchange (Unsal et al., 2006), electrophoresis (Hajduch et al., 2001), and affinity chromatography are also methods that are still used to this end (Wolschin et al., 2005; Raveendran et al., 2012). However, these traditional methods have disadvantages like deleterious effects on the protein activity induced by denaturation or complexation, higher costs, and setup complexity. DES-mediated liquid–liquid extraction has been shown previously to be a promising alternative method for protein purification in contrast to traditional methods resorting to the use of water or aqueous buffers in combination with organic solvents. Indeed, this last method is not desirable for protein purification because proteins can be easily denatured and lose partly or totally their activity as the essential water shell, for example, an enzyme can be disrupted by an excessively polar solvent. Keeping this in mind, an innovative protein purification method has been investigated that includes the use of ATPS. This system has been advantageously used to purify proteins because of its short purification time, capacity to ensure retention of the bioactivity, short phase separation time, high biocompatibility, and low toxicity (Gai et al., 2011; Oshima et al., 2010). DESs have been used as a part of ATPS to purify proteins as well (Table 5) (Saravanan et al., 2008; Zeng et al., 2016).

**TABLE 5:** Application of DES-based ATPS for protein purification.

ATPS	ATPS	Target protein	Protein purification rate (%)	Reference
Associated with DES	Betaine: glycerol: H_2_O (1: 2: 1) -K_2_HPO_4_	BSA	99.82	Li et al. (2016a)
	ChCl: glycerol (1: 1)-K_2_HPO_4_	BSA	98.71	Xu et al. (2015b)
	ChCl: glycerol (1: 1)-K_2_HPO_4_	Try	94.36	Xu et al. (2020)
	ChCl: urea (1: 2)-K_2_HPO_4_	R-phycoerythrin	92.60	Xu et al. (2020)
Other examples of purified protein by MSPE	PEG4000-MgSO_4_	BSA	82.68	Saravanan et al. (2008)
	Betaine-K_2_HPO_4_	BSA	90	Zeng et al. (2016)

Bridges et al. (2007) were the first to report an IL-based ATPS system in 2003. The advantages of this system were shorter separation time of proteins, low viscosity, and better extraction efficiency, which was quite an efficient transition from traditional ATPS methods and was a great contribution to a potentially scalable protein separation technique.

**FIGURE 3:**
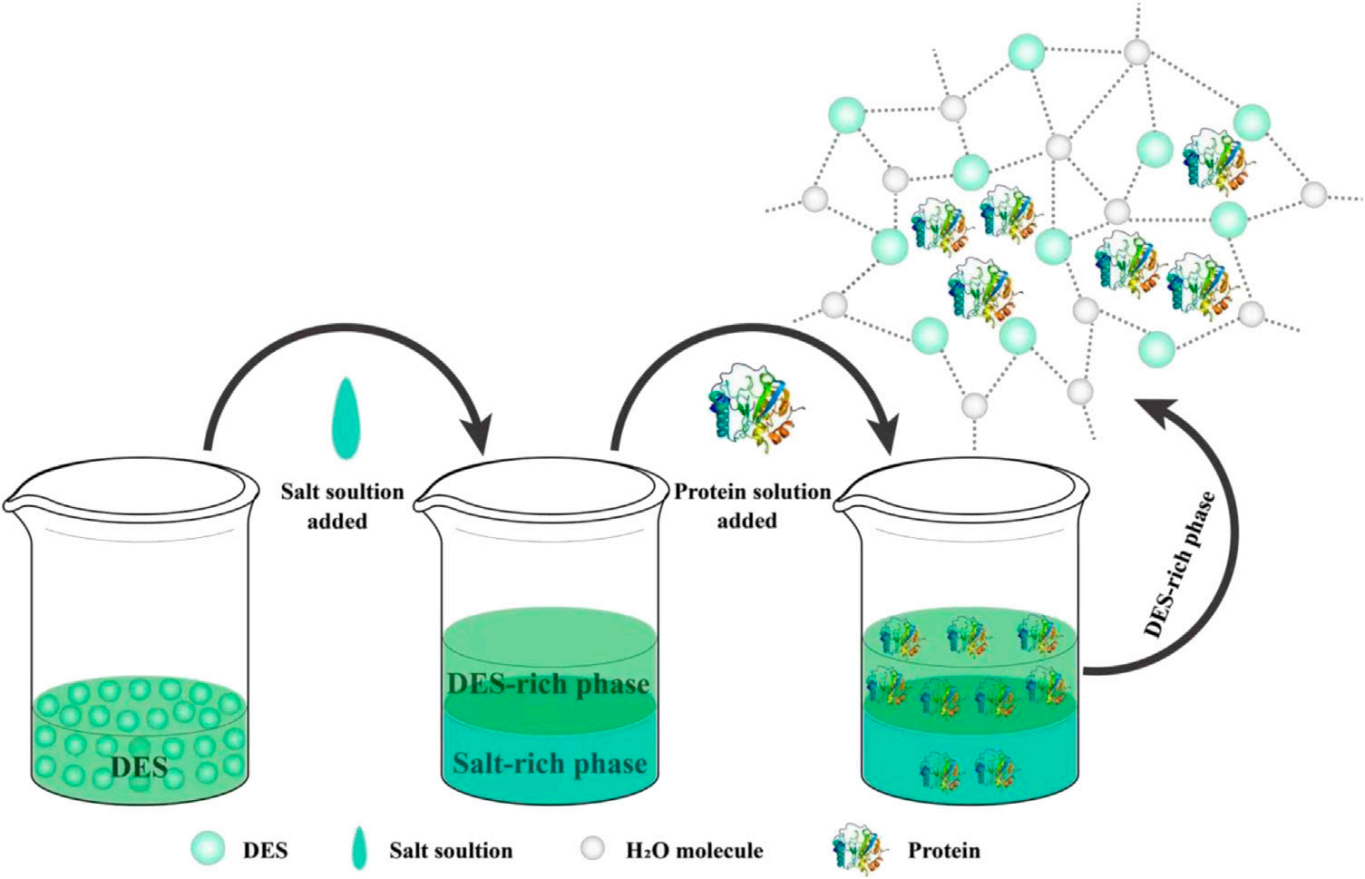
Application of DES-based ATPS for protein extraction and purification.

In this aim, Li et al. (2016b) prepared six different DESs by using betaine as the HBA and urea, methyl urea, glucose, sorbitol, glycerol, and ethylene glycol as HBD. These six DESs were used to extract and purify bovine serum albumin (BSA) using an ATPS, as depicted in Figure 3. They showed that DES in combination with ATPS can be used to extract and purify BSA from complex systems with efficiencies reaching 99.82% under optimized conditions. In that case, the betaine: urea combination was regarded as the most suitable DES.

Similar studies have shown the effects of DES on the purification of proteins by ATPS. Indeed, Xu et al. (2015a) used ChCl as the HBA and ethylene glycol, glycerol, glucose, and sorbitol as HBD to prepare four DESs for extraction and purification of proteins presenting catalytic activities such as BSA and trypsin in an ATPS. ChCl: glycerol in a 1:1 molar ratio was selected as the most suitable DES, and the conditions were optimized further by varying parameters such as pH, temperature, and time. Under the optimal extraction conditions, both BSA and trypsin were obtained with extraction rates of 98.71% and 94.36%, respectively.

Similarly, R-phycoerythrin, a red protein-pigment complex used for fluorescence-based detection with a broad absorption spectrum in the visible region was isolated from red algae. Xu et al. (2020) identified ChCl: urea (1:2) as the most suitable eutectic system for purification. Then, they established a ChCl: urea-K_2_HPO_4_ aqueous two-phase system to purify R-phycoerythrin with a recovery efficiency reaching 92.60% (Zeng et al., 2014; Xu et al., 2020).

## Promising innovation: Magnetic solid-phase extraction

Magnetic solid-phase extraction (MSPE) based on magnetic adsorbents can be seen as a complementary approach to ATPS in order to recover purely isolated proteins (Liu et al., 2012). In MSPE, magnetic adsorbents are dispersed into the sample solution, and these magnetic nanoparticles capture the analytes, that is, proteins, to be later separated directly from the sample matrix by means of an external magnetic field. Compared with standard solid-phase extraction methods, MSPE removes time-consuming steps such as centrifugation and filtration, ending up in a much-shortened processing time (Huang et al., 2015). In addition, analytes are easily eluted from the magnetic adsorbents to facilitate the recovery of both targeted molecules and adsorbents (Table 6) (Wen et al., 2016).

**TABLE 6:** Application of DES for protein purification using MSPE.

MSPE	MSPE	Target protein	Extraction capacity (mg g^−1^)	Reference
Associated with DES	Fe_3_O_4_@TiO_2_@ [ChCl][Xyl](1: 1)	Chy	347.8	Li et al. (2021)
	MB-NH_2_@CD@ [BeCh][Tri](1: 2)	Chy	549.87	Xu et al. (2020)
	Fe_3_O_4_-NH_2_@GO@ [ChCl] [glycerol] (1: 1)	BSA	44.59	Xu et al. (2015a)
	M-CNT@ N-[APTMAC][Xyl](1: 1)	BSA	225.15	Ni et al. (2019)
Other examples of purified proteins by MSPE	Fe@GO@Amino functional dicationic ionic liquid	BSA	89.7	Wen et al. (2016)
	Fe@GO	BSA	6.7	

**FIGURE 4:**
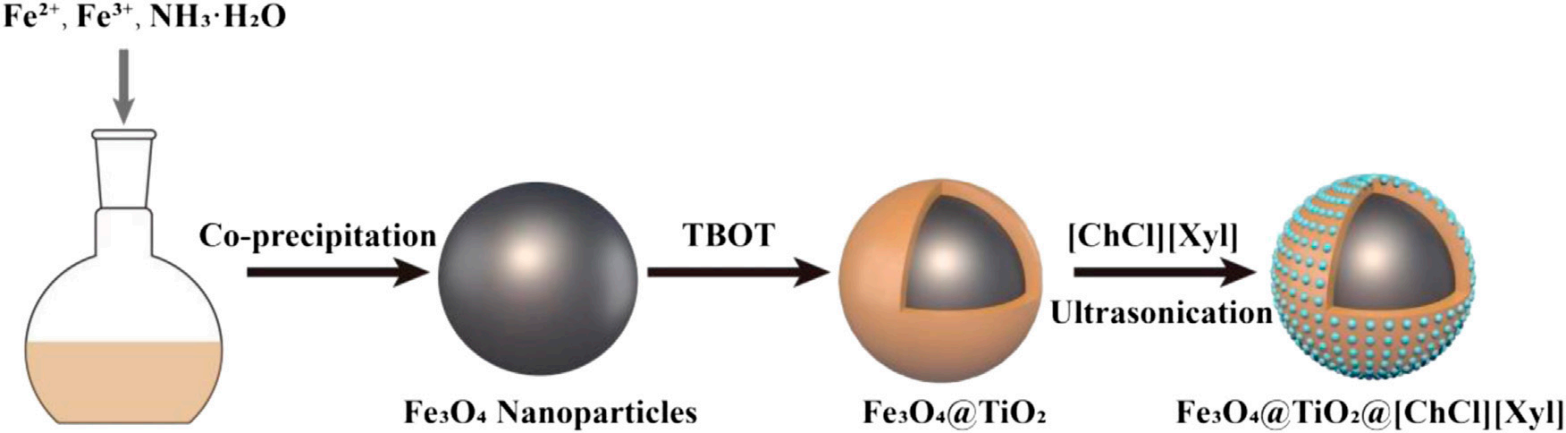
Magnetic titanium dioxide nanoparticles modified with DES.

Li et al. (2021) used four DESs such as ChCl: xylitol (1:1), ChCl: glycerol (1:1), tetrabutylammonium bromide: lactic acid (1:2), and benzyltributylammonium bromide: lactic acid (1:2) for the preparation of magnetic titanium dioxide nanoparticles modified by DES and applied them to MSPE of chymotrypsin (Figure 4). They found that Fe_3_O_4_@TiO_2_@[ChCl][Xylitol] gave the best results among the four DES-modified particles, when using 10% sodium dodecyl sulfate-acetic acid as the eluent to reach an extraction efficiency of 85.9%. In addition, Fe_3_O_4_@TiO_2_@[ChCl][xylitol] particles maintained excellent extraction efficiency toward chymotrypsin after six cycles of use (Li et al., 2021). MSPE in combination with DES is a novel approach for protein purification, allowing superior recovery of protein. This method permits to reach higher protein purity while using mild processing conditions that do not affect the functions of the targeted molecules. Therefore, it is a promising strategy that could play a considerable role in future protein purification processes (Huang et al., 2015).

## Conclusion

DES are a class of greener alternative solvents that have attracted considerable attention in biorefinery research due to their biodegradability, sustainability, low toxicity, and facile preparation. In addition to their numerous applications in biocatalysis, they are currently used as greener solvents but nonetheless efficient extraction and separation media for the recovery of animals and plants as protein-rich renewable biomass and thus provides new perspectives to biorefineries. Despite technical limitations intrinsic to DESs, such as viscosity, they have been shown to be a promising alternative solvent for extraction of a wide variety of proteins and can be compatible with various raw biomasses. It is to foresee that their implementation in industrial refineries could solve technical and methodological issues that are currently encountered in the food industry such as the valorization of wastes or the volatility of currently used organic solvents. Selection of isomeric analogs of HDB, the presence of water during DES formation, and the structure–function relationship existing between extracted proteins and DESs need to be kept in mind. Therefore, optimization of DES preparation in order to be simple and environmentally friendly needs to be a priority when designing protein extraction processes when starting from either animal or plant sources. Further research should facilitate the use of DESs in the sustainable development of protein purification in the food industry by notably demonstrating proof of their scalability.
